# Botanical Drug Puerarin Promotes Neuronal Survival and Neurite Outgrowth against MPTP/MPP^+^-Induced Toxicity via Progesterone Receptor Signaling

**DOI:** 10.1155/2020/7635291

**Published:** 2020-10-17

**Authors:** Yingke Zhao, Jia Zhao, Xiuying Zhang, Yuanyuan Cheng, Dan Luo, Simon Ming-Yuen Lee, Lixing Lao, Jianhui Rong

**Affiliations:** ^1^School of Chinese Medicine, Li Ka Shing Faculty of Medicine, The University of Hong Kong, Pokfulam, Hong Kong; ^2^Department of Chinese Medicine, The University of Hong Kong Shenzhen Hospital, Shenzhen, China; ^3^State Key Laboratory of Quality Research in Chinese Medicine, Institute of Chinese Medical Sciences, University of Macau, Macau, China; ^4^The University of Hong Kong Shenzhen Institute of Research and Innovation (HKU-SIRI), Shenzhen, China

## Abstract

**Background:**

Progesterone receptor (PR) modulates neuroprotective and regenerative responses in Parkinson's disease and related neurological diseases.

**Objectives:**

The present study was designed to determine whether botanical drug puerarin could exhibit neuroprotective and neurorestorative activities via PR signaling.

**Methods:**

The neuroprotective and neurotrophic activities of puerarin were investigated in MPTP-lesioned mice and MPP^+^-challenged primary rat midbrain neurons. Rotarod performance test and tail suspension test were used to assess motor functions. Tyrosine hydroxylase (TH) and PR were determined by immunostaining, Western blotting, and luciferase reporter assays. Neurite outgrowth was assessed by fluorescence staining and immunostaining.

**Results:**

Puerarin effectively ameliorated the MPTP-induced motor abnormalities in MPTP-lesioned mice and protected primary rat midbrain neurons against MPP^+^-induced toxicity via PR signaling although progesterone exhibited the neuroprotection. PR antagonist mifepristone (RU486) diminished the neuroprotection of puerarin in MPTP-lesioned mice and MPP^+^-induced primary rat midbrain neurons. Moreover, puerarin promoted the differentiation of primary rat midbrain neurons and potentiated NGF to induce neuritogenesis in PC12 cells. RU486 and PR-siRNA could inhibit the effect of puerarin. Puerarin and progesterone could enhance the PR promoter.

**Conclusion:**

Puerarin attenuated MPTP- and MPP^+^-induced toxicity and potentiated neurite outgrowth via PR. These results suggested that puerarin may become an alternative hormone for suppressing MPTP- and MPP^+^-induced toxicity in neurodegenerative diseases.

## 1. Introduction

Parkinson's disease (PD) is the second most common neurodegenerative disease worldwide and characterized by motor dysfunctions (e.g., tremor, rigidity, and dyskinesia) as well as some other nonmotor symptoms (e.g., cognition deficit and depression) [1]. The pathological change in PD patients is hallmarked by the progressive loss of dopaminergic neurons in substantia nigra compacta (SNpc) [2]. Levodopa is a commonly accepted drug for the treatment of PD although the unavoidable side effects and instability are documented after long-term use [3]. Therefore, it is pressingly needed to discover new neuroprotective and neuroregenerative small molecules to meet the clinical demand.

Clinical evidence shows that men suffer from higher prevalence and incidence of PD than women, suggesting the neuroprotective role of sex hormones in the brain [4, 5]. Estrogens (e.g., 17*β*-estradiol and estradiol) showed neuroprotective potential to diminish the nigrostriatal degeneration in mouse model of PD [6, 7]. However, the feminizing effects of estrogens limit its medical use in the treatment of PD. On the other hand, progesterone is free of feminizing effects and could be available for both men and women [4]. Although progesterone is classically recognized as an endogenous steroid from ovary, Pomata et al. found that progesterone could be synthesized from cholesterol in the central nervous system [8]. Several studies showed that progesterone played the neuromodulatory and neuroprotective roles in PD [9, 10]. Presumably, progesterone exhibits neuroprotective effects via interacting progesterone receptor (PR) [11]. PR exists in two isoforms: PR-A and PR-B, which are transcribed from two different promoter regions of single gene and expressed in the brain [12]. PR-B is a stronger transcriptional activator compared with PR-A [13]. Interestingly, PRs are recognized as therapeutic target for the control of neurodegeneration [14].

**Figure 1:**
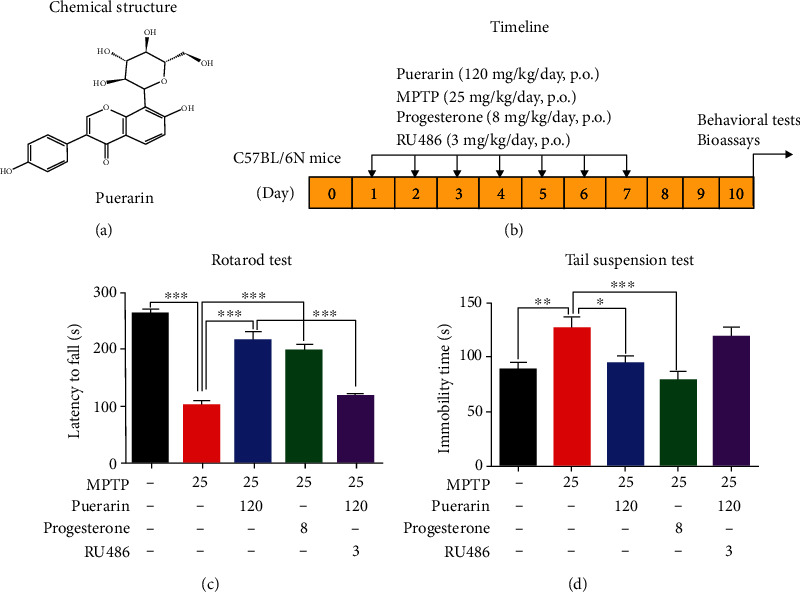
Puerarin ameliorated behavioral impairments in MPTP-lesioned mice. (a) Chemical structure of puerarin. Chemical structure was generated by ChemSketch (http://www.acdlabs.com/home/). (b) Design for animal experiments. C57BL/6N mice were randomly divided into five groups: control, MPTP, MPTP+Puerarin, MPTP+Progesterone, and MPTP+Puerarin+RU486. Following treatments, the mice were assessed for behavioral impairments and biomarker expression. (c) Rotarod performance test. Animals were evaluated for the riding time. (d) Tail suspension test. Animals were assessed for the immobility time. The results of behavioral tests were presented as mean ± SEM (*n* = 10) and analyzed by one-way ANOVA followed by Dunnett's test. ^∗^*p* < 0.05, ^∗∗^*p* < 0.01, ^∗∗∗^*p* < 0.001.

Herbal medicines are well documented for the chemical compositions of stilbenoids, flavonoids, catechols, and terpenes and the potential activities against neurodegeneration [15, 16]. Puerarin (Figure 1(a)) is a unique C-glycosylated isoflavone from the herb *Radix Puerariae lobatae* [17]. We previously demonstrated that puerarin markedly reduced neurotoxicity of 6-hydroxydopamine (6-OHDA) and MPTP, *in vitro* and *in vivo*, respectively [18, 19]. As far as the mechanism is concerned, puerarin might mimic estrogen to protect the nigral neurons from apoptosis [20, 21]. On the other hand, puerarin could recover the decrease in progesterone levels in rats that suffered from chronic stress and posttraumatic stress disorder [22, 23]. Puerarin disrupted the implantation of the rat uterus by inhibiting PR [24]. Thus, the present study was designed to determine whether puerarin could exhibit neuroprotective and neurorestorative activities via PR signaling.

## 2. Materials and Methods

### 2.1. Biochemical Reagents

Antibody against tyrosine hydroxylase (TH) was purchased from Merck Millipore (Billerica, MA, USA). Antibodies against GAPDH and Alexa Fluor 594-conjugate anti-rabbit IgG antibody were purchased from Cell Signaling Technology (Boston, BA, USA). Mouse antigrowth-associated protein 43 (GAP43) antibody and rabbit anti-PR antibody were purchased from Santa Cruz Biotechnology Inc. (Santa Cruz, CA, USA). Protein assay dye reagent concentrate was purchased from Bio-Rad Labs (Hercules, CA, USA). Enhanced chemiluminescence (ECL) detection reagent was purchased from GE Healthcare (Uppsala, Sweden). 3,3-N-diaminobenzidine tetrahydrochloride (DAB) substrate kit was obtained from Dako Corporation (Carpintera, CA, USA). PR siRNA, HiPerFect transfection reagent, and Effectene Transfection Reagent were purchased from Qiagen (Hilden, Germany). The luciferase reporter vectors (i.e., 2xPRE-TK-Luc) were obtained from Addgene (Cambridge, MA, USA). pRL-TK vector encoding Renilla luciferase and dual-luciferase reporter assay system was purchased from Promega (Madison, USA). The neurite outgrowth staining kit was purchased from Invitrogen (Carlsbad, CA, USA). HRP-conjugated anti-rabbit IgG secondary antibody, MPTP, MPP^+^ iodide, NGF-2.5S, Hoechst 33342, and propidium iodide (PI) were purchased from Sigma-Aldrich (St. Louis, MO, USA). Puerarin and RU486 (mifepristone) were purchased from Yick-Vic Chemicals & Pharmaceuticals (Shatin, Hong Kong).

### 2.2. Animals and Drug Treatment

The experimental procedures were approved by the Committee on the Use of Live Animal in Teaching and Research (CULATR No. 4046-16), University of Hong Kong. The animal experiments were outlined in Figure 1(b). C57BL/6N male mice (7-8 weeks old, 21-25 g) were provided by the Laboratory Animal Unit on the campus and housed under a controlled temperature (25 ± 2°C) and humidity (55 ± 10%) condition in a 12-12 h light/dark cycle at the animal unit. For drug treatment, puerarin and progesterone were dissolved in saline containing 50% 1,2-propylene glycol, and the solutions were sterilized via 0.22 *μ*m filters from Pall Corporate (Port Washington, NY, USA). Prior to use, RU486 was freshly prepared in saline containing 1% Tween-80 with 30-second sonication, while MPTP was dissolved in saline. Following the regulation of the university safety office, when MPTP was used, mice were isolated in carcinogen suite for additional 3 days after the final treatment. Thereafter, mice were moved to the conventional facility for behavioral tests and bioassays.

### 2.3. Animal Experimental Design

The doses and delivery of different drugs were decided based on previous publications [9, 18, 25]. Briefly, mice were firstly randomly divided into five groups (*n* = 10): control, MPTP, MPTP+Puerarin, MPTP+Progesterone, and MPTP+Puerarin+RU486. Mice were treated with MPTP (25 mg/kg/day via intraperitoneal injection for 7 days while animals in the control group were injected the equal volume of saline. At the time point of 4 hours prior to daily MPTP injection, puerarin (120 mg/kg/day) or progesterone (8 mg/kg/day) was administered to the mice in the indicated group via oral gavage, while mice in the control and MPTP groups were injected the same volume of vehicle. For RU486 administration, mice were intraperitoneally injected with RU486 (3 mg/kg/day) or vehicle at 30 min prior to puerarin treatment for 7 days.

### 2.4. Rotarod Performance Test

Mice were evaluated for motor impairments with a rotarod apparatus (Harvard apparatus, Holliston, MA, USA) as described [26, 27]. In brief, at 3 days after last dosage of MPTP treatment, mice were subjected to behavioral assessments. On the day for behavioral tests, animals were firstly pretrained for 3 sessions prior to the assessment and placed on the lane (constant 15 rpm) for 5 min. The dropping time was automatically recorded for each mouse. Three independent tests were performed with each mouse at 1 h intervals.

### 2.5. Tail Suspension Test

Tail suspension test was carried out at 4 hours after rotarod test as described [26]. Briefly, mice were suspended by fixing the tail to the pole with adhesive tape. The distance between the mouse's nose and the bottom was 20-25 cm, and the test time was 6 min. Mice were monitored by video during the process, while the results were analyzed by the observers after the behavior tests. The immobility time of each mouse within 6 min was measured for assessing the motor functions.

### 2.6. Immunohistochemistry Staining

The expression of TH in the midbrain was detected by immunohistochemical staining as previously described [27]. In brief, mice were transcardially perfused sequentially with saline and 4% paraformaldehyde under anesthetic condition. The brains were collected and fixed in 4% paraformaldehyde overnight at 4°C. Following immersed in a 30% sucrose containing 4% paraformaldehyde, the brains were embedded in Tissue-Tek O.C.T. Compound (Sakura, USA) and stored at -80°C. The cryosections were cut into serial coronal sections at a thickness of 30 *μ*m on a freezing microtome (Model CM-1850, Leica, Germany). After thawed at room temperature for 1 hour, the cryostat sections were heated in antigen retrieval buffer, immersed in 3% hydrogen peroxide solution to quench the endogenous peroxidase, and subsequently blocked with 5% goat serum to avoid nonspecific staining. For TH detection, the brain sections were probed with anti-TH primary antibody overnight at 4°C. After excessive wash, the bound antibodies were detected with HRP-conjugated goat anti-rabbit secondary antibody for 1 hour at room temperature. The horseradish peroxidase was then assayed with 3,3-N-diaminobenzidine tetrahydrochloride (DAB) substrate kit from Dako (Carpintera, CA, USA). The slices were counterstained with hematoxylin and imaged under an Olympus microscope (Olympus Corp, Tokyo, Japan). The number of TH^+^ neurons in each section was counted within three nonoverlapping views at tenfold magnification.

### 2.7. Western Blotting Analysis

The expression of indicated biomarkers was examined by Western blotting as described [26]. Briefly, midbrain tissues, primary midbrain neurons, and PC12 cells were lysed in RIPA buffer containing 1x protease inhibitor cocktail. The proteins were recovered by centrifugation at 13,000 rpm for 15 min at 4°C. The concentration of proteins was determined with the protein assay dye reagent from Bio-Rad (Hercules, CA, USA). Proteins (70 *μ*g for tissue, 30 *μ*g for cells) were resolved on 10% SDS-polyacrylamide gels and transferred onto PVDF membranes. After 2-hour incubation in 5% BSA in Tris buffer containing 1% Tween-20 (TBS-T), the membranes were incubated with primary antibodies overnight at 4°C and then detected with HRP-conjugated goat anti-rabbit IgG secondary antibodies for another 3 hour at 4°C. The blots were visualized with Amersham™ ECT™ detection reagent from GE Healthcare (Uppsala, Sweden).

### 2.8. PC12 Cell Culture

Rat pheochromocytoma PC12 cell line was obtained from American Type Culture Collection (Manassas, VA, USA) and cultured in Dulbecco's modified Eagle's medium (DMEM) containing 10% horse serum (HS), 5% fetal bovine serum (FBS), and 1% penicillin/streptomycin (Invitrogen, Carlsbad, CA, USA) in incubators with 37°C and 5% CO_2_ atmosphere. For drug treatment, the cells were cultured to the density of 70-80% and subsequently treated with indicated drugs.

### 2.9. Primary Culture of Rat Midbrain Neurons

Primary rat midbrain neurons were isolated from 17-day-old Sprague-Dawley (SD) rat embryos as previously described [27]. Briefly, 1 day before the isolation of the midbrain neurons, 6-well plates were precoated with poly-D-lysine. The midbrain neurons were dissociated by using trypsin and DNase. After centrifugation, freshly isolated neurons were seeded onto six-well plates at a density of 5 or 8 × 100000 cells/ml and cultured in Neurobasal® medium containing 2% B27 supplement from Thermo Fisher Scientific Inc. (Waltham, MA, USA) for 3 or 7 days prior to drug treatment.

### 2.10. Hoechst 33342/Propidium Iodide Staining

Freshly isolated midbrain neurons were seeded on 6-well-plate at the density of 1 × 10^6^ cells/well and cultured for 7 days. The neurons were treated with puerarin, progesterone, RU486, and MPP^+^, alone or in combination for 24 h. To stain the dead cells, 5 *μ*M Hoechst 33342 and 5 *μ*M PI were added to each well. The neurons were visualized under a Zeiss fluorescence microscope from Carl Zeiss Company (Jena, Germany).

### 2.11. Immunofluorescence Staining

The expression of PR in the brains was examined by the immunofluorescence staining as previously described [5, 26, 28]. Following antigen retrieval, the slides were immersed in 0.5% Triton X-100 in PBS for 30 mins and blocked in 5% goat serum for 2 h. The sections were probed with polyclonal antibody against PR overnight at 4°C, washed with PBS for 3 times, and detected with Alexa Fluor 594-conjugated rabbit IgG secondary antibody. The cell nuclei were stained with 4′-6-diamidino-2-phenylindole (DAPI) for 10 min. After the slides were mounted with coverslips, the images were then acquired under a Zeiss LSM 780 confocal microscopy from Carl-Zeiss Company (Jena, Germany) with 20x objective lens.

On the other hand, GAP43 was detected by immunostaining as previously described [29]. After the drug treatment with puerarin and RU486, alone or in combination for 48 h, the neurons were fixed in 4% paraformaldehyde, incubated in 0.5% Triton X-100 for 30 min to permeabilize the cells, and then blocked in 5% normal goat serum for 2 h at room temperature. The cells were then incubated with anti-GAP43 primary antibodies at 4°C overnight, and the bound antibodies were detected with Alexa Fluor 594 anti-rabbit IgG secondary antibody for 2 h at room temperature. After the cells were washed with PBS for three times, the cell nuclei were then stained with DAPI for 10 min. The fluorescence images were visualized under fluorescence microscope (Carl Zeiss, Jena, Germany). The neurite length of GAP-43^+^ neurons was automatically detected by ImageJ software (http://imagej.nih.gov).

### 2.12. siRNA-Mediated Silencing of PR Expression

The expression of PR in PC12 cells was transiently silenced with PR-specific siRNAs as previously described [28]. Briefly, PC12 cells were grown on poly-L-lysine precoated coverslips and then transfected with specific siRNAs using HiPerFect Transfection Reagent according to the manufacturer's protocol. After 24 h incubation, the transfected cells were treated with puerarin and NGF, alone or in combination, for 72 h. The cells were then stained with neurite outgrowth staining kit and analyzed for PR expression by Western blotting with specific antibodies.

### 2.13. Assay of Neurite Outgrowth

Neurite outgrowth was assessed by commercial neurite outgrowth assay kit as described [18]. In practice, PC12 cells were transfected with specific siRNAs and subsequently treated with puerarin and NGF, alone or in combination, for 72 h. The cells were stained by neurite outgrowth assay kit according to the manufacture's instruction. The neurites were imaged under a Zeiss fluorescence microscope. The average length of neurites was determined by Image J software (https://imagej.nih.gov/ij/).

### 2.14. Transfection and Luciferase Reporter Assays

The promoter activities of PR were assayed using promoter-reporter system as previously described [30]. Briefly, PC12 cells were seeded in 96-well plates at the density of 1 × 10^5^ cells/well, cultured to the cell density of 70-80% and transiently transfected with 0.1 *μ*g 2xPRE-TK-Luc and 0.05 *μ*g pRL-TK. The transfection was continued for 6 h using Transfectene (Qiagen) according to the manufacturer's instruction. At 24 h after transfection, the cells were treated with puerarin and progesterone for 24 h. The promoter activity was then assayed with the Dual-Luciferase Reporter System according to the manufacturer's protocol while the luciferase activity was monitored on a Clariostar microplate reader from BMG Labtech (Ortenberg, Germany). The results were expressed as luciferase activity after normalization to Renilla activity.

### 2.15. Molecular Docking

The crystal structure (PDB: 3D90) of human PR was downloaded from RCSB PDB website (http://www.rcsb.org/pdb). The chemical structures of the ligand molecules (i.e., puerarin and progesterone) were generated by the ChemBioDraw Ultra 12.0 software. The protein-ligand interactions were simulated by the AutoDock Vina in PyRx-virtual screen tool package. Prior to docking, all water molecules were removed from PR structure. The docking results were analyzed by the Discovery Studio Visualizer software.

### 2.16. Statistical Analysis

The results were represented as mean ± SEM for behavioral tests and mean ± SD for other experiments. The statistical significance was determined by one-way analysis of variance (ANOVA) followed by Dunnett's test with GraphPad Prism 6 (La Jolla, CA, USA). A *p* value of <0.05 was considered as statistically significant.

## 3. Results

### 3.1. Puerarin Ameliorated Behavioral Impairments in MPTP-Lesioned Mice

To validate the *in vivo* neuroprotective activity of puerarin, we firstly employed rotarod performance test and tail suspension test to determine the effects of puerarin on the behavioral deficits in MPTP-treated mice. In practice, the animals were sequentially evaluated for motor disability by rotarod performance test and the stress response by tail suspension test while two tests were performed in a 4-hour interval. As shown in Figure 1(c), MPTP reduced the time for mice to stay on the rod compared with control mice. Both puerarin and progesterone effectively helped mice to ride on the rotarod for longer time compared with untreated MPTP mice. PR inhibitor RU486 diminished the beneficial effects of puerarin in rotarod performance test. As shown in Figure 1(d), on the other hand, tail suspension test also confirmed that MPTP prolonged the immobility time of mice whereas puerarin and progesterone markedly reduced the immobility time of mice. RU486 reversed the beneficial effects of puerarin.

### 3.2. Puerarin Enhanced the Survival of Dopaminergic Neurons against MPTP Neurotoxicity

**Figure 2:**
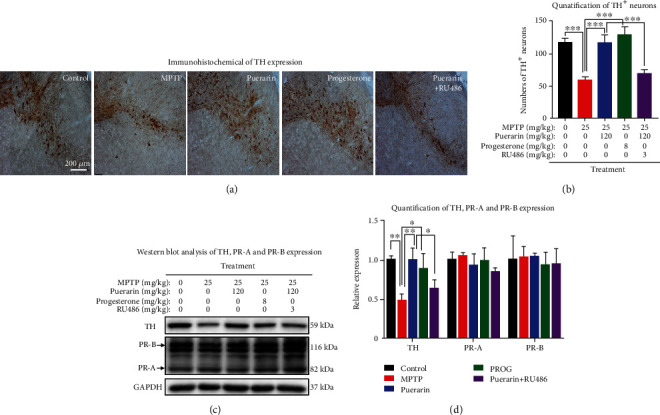
Puerarin protected dopaminergic neurons from MPTP-induced damage in mice. (a) Immunohistochemical staining of TH expression. The frozen brain sections were analyzed by immunohistochemical staining with anti-TH antibody. The images were captured under an Olympus microscope (Tokyo, Japan). (b) Quantification for TH^+^ neurons. The number of TH^+^ neurons in each group (*n* = 3) were counted. ^∗∗∗^*p* < 0.001. (c) Western blot analysis of TH, PR-A, and PR-B expression. Midbrain tissues were collected and analyzed by Western blotting with antibodies against TH, PR-A, and PR-B, whereas GAPDH was analyzed as control. Representative blots for each group were shown. (d) Quantification of TH, PR-A, and PR-B expression. Western blots (*n* = 3) in (c) were determined by a densitometric method. ^∗^*p* < 0.05, ^∗∗^*p* < 0.01.

To evaluate the *in vivo* neuroprotective activity of puerarin, dopaminergic neurons was examined by detecting the expression of TH in MPTP-treated mice. As shown in Figures 2(a) and 2(b), MPTP dramatically reduced TH expression as the indicator for TH-positive dopaminergic neurons in SNpc, whereas both puerarin and progesterone effectively halted the loss of dopaminergic neuron. RU486 attenuated the beneficial effects of puerarin. On the other hand, the expression of TH was also assessed by Western blotting with specific antibody. The results in Figures 2(c) and 2(d) essentially verified the neuroprotective effects of puerarin on dopaminergic neurons against MPTP neurotoxicity and the inhibitory effects of RU486 on the activity of puerarin.

### 3.3. Puerarin Did Not Change the Expression of PR in MPTP-Lesioned Mice

**Figure 3:**
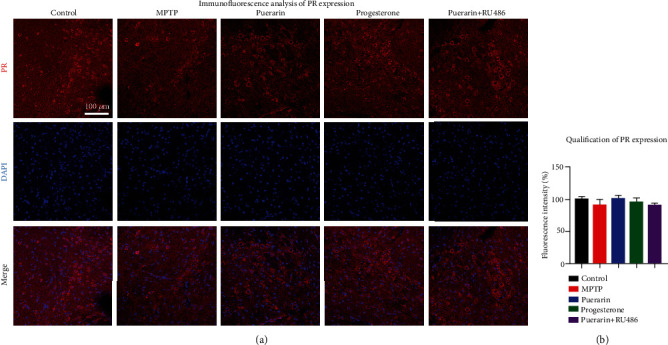
Immunofluorescence analysis of PR expression. (a) Detection of PR expression. The model of MPTP-induced parkinsonism was treated with puerarin (120 mg/kg/day), progesterone (8 mg/kg/day), or RU486 (3 mg/kg/day), as indicated. Midbrain cryosections were probed with primary anti-PR antibody and visualized with Alexa Fluor 594-conjugated secondary antibody, whereas the cell nuclei were stained with DAPI. The substantia nigra areas were examined under a Zeiss LSM 780 confocal microscopy. Representative images with 20x amplification were shown. Scale bar, 100 *μ*m. (b) Quantitative analysis of PR levels. Fluorescence intensity of PR staining in the midbrain was quantified by a densitometric method and expressed in mean ± SD (*n* = 3).

To clarify why RU486 could antagonize puerarin, we employed Western blotting and fluorescence immunostaining techniques to determine the effect of puerarin on the expression of PR in the MPTP-treated mice. Western blotting results in Figures 2(c) and 2(d) did not show much difference in the levels of PR expression between different treatment groups. The immunofluorescence staining results in Figure 3 further confirmed that the levels of PR expression in the brain were not changed under the experimental conditions.

### 3.4. Puerarin Exerted Neuroprotective Properties in Primary Midbrain Neurons

**Figure 4:**
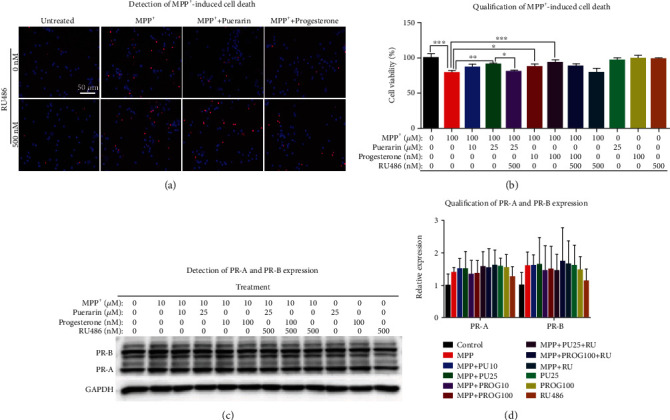
Puerarin enhanced the survival of primary midbrain neurons against MPP^+^-induced neurotoxicity. (a) Detection of MPP^+^-induced cell death. Primary midbrain neurons were treated with MPP^+^, puerarin, progeresterone, and RU486 as indicated. Cell viability was assessed by Hoechst 33342 and PI staining. The representative images were shown. (b) Quantification of neuronal viability. Hoechst- and PI-positive cells were counted under a Zeiss fluorescence microscope (Carl Zeiss, Jena, Germany). ^∗^*p* < 0.05; ^∗∗^*p* < 0.01; ^∗∗∗^*p* < 0.001. (c) Western blot analysis of PR-A and PR-B expression. Primary midbrain neurons were treated with MPP^+^, puerarin, progeresterone, and RU486 as indicated. The expression of PRs was analyzed by Western blot analysis with specific antibodies, while GAPDH was analyzed as the loading control. Representative blot was shown. (d) Quantification of PR-A and PR-B expression. Western blots (*n* = 3) in (c) were determined by a densitometric method. ^∗^*p* < 0.05.

To verify the *in vitro* neuroprotective activities of puerarin, we employed fluorescence dye PI to detect MPP^+^-induced cell death in primary rat midbrain neurons. Primary rat midbrain neurons were freshly isolated for each experiment. Following 7-day culture in complete growth medium, the neurons were challenged with MPP^+^, and the neuronal viability was assessed by PI/Hoechst 33342 staining. As shown in Figures 4(a) and 4(b), 100 *μ*M MPP^+^ dramatically caused cell death, whereas puerarin (25 *μ*M) and progesterone (10 nM or 100 nM) effectively enhanced the survival of neurons against MPP^+^-induced neurotoxicity. RU486 effectively prohibited the neuroprotective activity of puerarin and, to a lesser extent, affected the activity of progesterone. On the other hand, the expression of PR in primary neurons was also determined by Western blot analysis. As shown in Figures 4(c) and 4(d), the expression of PR did not vary much between the treatment groups.

### 3.5. Puerarin Promoted Neurite Outgrowth via PR Signaling

**Figure 5:**
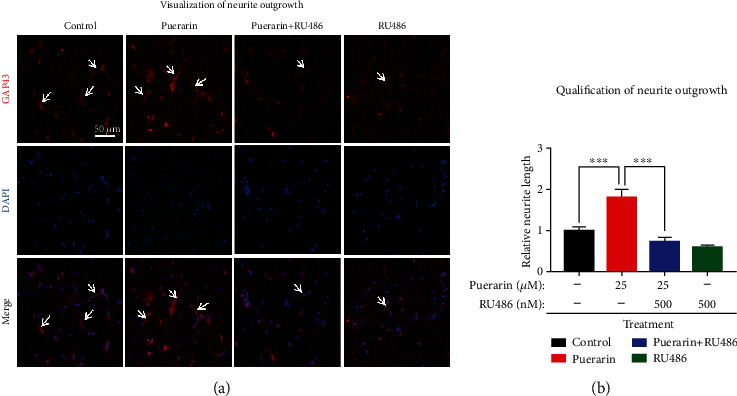
Puerarin promoted neurite outgrowth in primary midbrain neurons. (a) Visualization of neurite outgrowth. Primary midbrain neurons were treated with puearin and RU486 as indicated. Following immunofluorescence staining with anti-GAP43 antibody and DAPI, the cells were examined under a fluorescence microscope from Carl Zeiss (Jena, Germany). Scale bar, 50 *μ*m. (b) Quantification of neurite outgrowth. The neurite length was quantified by using ImageJ (http://imageJ.nih.gov). Neurites from three nonoverlapping fields for each slice were recorded and analyzed for (a). ^∗∗∗^*p* < 0.001.

To further determine whether puerarin exhibits neurotrophic activities in a PR-dependent manner, we evaluated the effects of puerarin on neurite outgrowth in primary midbrain neurons and PC12 cells. As shown in Figures 5(a) and 5(b), puerarin greatly promoted the neurite extension in primary midbrain neurons. RU486 abolished the neurotrophic effect of puerarin so that puerarin did not increase the neurite length in the presence of RU486 relative to the control group.

**Figure 6:**
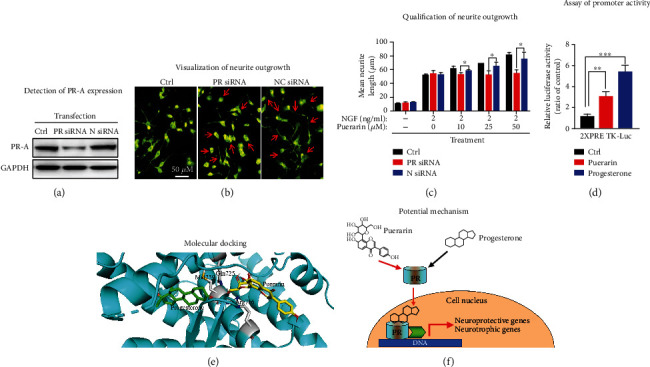
Puerarin promoted neurite outgrowth via PR signaling. (a) siRNA-mediated silencing of PR in PC12 cells. PC12 cells were transiently silenced by PR siRNAs and negative control siRNAs. The expression of PR was determined by Western blot analysis with anti-PR antibody. Representative blots were shown. (b) Visualization of neurite outgrowth. After siRNA transfection, PC12 cells were treated with puerarin and NGF for 72 h. Neurite outgrowth was determined by a commercial neurite staining kit and analyzed under a fluorescence microscope from Carl Zeiss (Jena, Germany). The representative images were shown. Scale bar, 50 *μ*m. (c) Quantification of neurite outgrowth. Neurites from three nonoverlapping fields for each slice were recorded and analyzed. ^∗^*p* < 0.05, ^∗∗^*p* < 0.01, ^∗∗∗^*p* < 0.001. (d) Luciferase assay for promoter activity. PC12 cells were transiently transfected with 2xPRE-TK-Luc and pRL-TK and cultured for 24 h. The cells were subsequently treated with puerarin and progesterone for 24 h. The promoter activity was assayed with the Dual-Luciferase Reporter System under a Clariostar microplate reader from BMG Labtech (Ortenberg, Germany). Results were expressed as luciferase activity and normalized to Renilla activity for three independent experiments. ^∗∗^*p* < 0.01, ^∗∗∗^*p* < 0.001. (e) Molecular docking of puerarin and progesterone. Puerarin and progesterone were docked into PR structure (PDB: 3D90) by AutoDock Vina. (f) Potential mechanism. Puerarin may interact with PR and activate the transcriptional factor activity of PR to induce neuroprotective genes and neurotrophic factors.

To determine the role of PR in the neurotrophic activities of puerarin, on the other hand, we successfully generated a PR-silenced PC12 cell model by transiently transfecting specific siRNAs for evaluating the effects of puerarin on neurite outgrowth. As shown in Figure 6(a), PR-specific siRNAs effectively downregulated PR expression whereas the negative control siRNAs did not alter PR expression. Upon transfection with PR siRNAs, as shown in Figures 6(b) and 6(c), the transfected cells failed to generate neurites in response to puerarin (10, 25, and 50 *μ*M) whereas PC12 cell transfected with the negative control siRNA group responded to puerarin for neurite outgrowth in a highly similar fashion compared with the untransfected cells.

To clarify whether puerarin could activate PR-mediated transcriptional activity, we introduced the promoter-reporter construct carrying PR-binding DNA sequence into PC12 cells. In the experiments, PC12 cells were transfected with plasmid DNA construct 2xPRE-TK-Luc. As shown in Figure 6(d), puerarin and progesterone markedly increased the PR-mediated transcriptional activity. Based on molecular docking as shown in Figure 6(e), puerarin and progesterone bind to the different sites of PR with the binding energy of -8.2 kcal/mol for puerarin and -10.1 kcal/mol for progesterone, respectively. Puerarin appeared to be a moderate to strong binder, whereas progesterone is a high affinity ligand.

## 4. Discussion

Early diagnosis and early intervention are equally important to maintain the life quality of PD patients [31, 32]. While the efficacy of the existing clinical drugs is controversial, natural product flavonoids hold promise for halting the progression of the disease and restoring the neuronal functions in PD patients [17]. As a key example, puerarin is extensively evaluated for the neuroprotective effects in different PD models [33–35]. We previously demonstrated several indirect mechanisms underlying the neuroprotective and neurotrophic effects of puerarin [18, 19]. The present study focused on the question of whether puerarin could modulate PR signaling to exhibit neuroprotective and neurotrophic activity.

The progesterone-PR signaling pathway is initially known for its important regulatory role in female reproduction and the link to the progression of breast cancer [13]. Meanwhile, a few earlier studies reported that endogenous progesterone and PR could exhibit cerebroprotection in stroke [36]. Progesterone is now known as an important neurosteroid for its capacity of protecting neurons and promoting neuronal repair in PD and traumatic brain injury [9, 37]. The neuroprotective potential of progesterone was validated to be PR-dependent in MPTP-induced PD models [4, 38]. These results highlight that PR signaling is a potential therapeutic target for the treatment of neurodegeneration [14]. Progesterone induces genomic and nongenomic signals through classical intracellular PRs, the seven transmembrane domain 7TMPRb, and the membrane-associated PR component 1 (PGRMC1) [39]. Progesterone directly interacts with PR and promotes the translocation of PR into cell nuclei, whereas PR functions as a transcriptional factor to regulate the expression of genes for various functions including neuroprotection and neuronal restoration [40].

On the other hand, puerarin is classically tested for its estrogenic activity in the animal models [41]. In the present study, we found that puerarin (120 mg/kg) could reduce MPTP-induced motor dysfunctions to the similar extent to progesterone (8 mg/kg) (Figures 1(c) and 1(d)). RU486 effectively antagonized the beneficial effect of puerarin on MPTP-induced behavioral impairments. Based on the immunohistochemical analysis for TH expression, puerarin (120 mg/kg) and progesterone (8 mg/kg) enhanced the survival of dopaminergic neurons against MPTP-induced neurotoxicity to a similar extent. Again, RU486 abolished the neuropotective effect of puerarin (Figures 2(a) and 2(b)). The assay of neuronal viability also confirmed the neuroprotective activity of puerarin and the antagonizing effect of RU486 (Figures 4(a) and 4(b)). Furthermore, puerarin effectively promoted neurite growth in primary rat midbrain neurons, whereas RU486 abolished the neurotrophic activity of puerarin. Importantly, puerarin did not alter the *in vivo* and *in vitro* expression of PR (Figures 2(c), 2(d), 3, 4(c), and 4(d)). Taken together, these results suggested that puerarin might be able to interact with PR or some signaling molecules in PR signaling pathway. In the present study, therefore, we examined the role of PR in the beneficial effects of puerarin. Our strategy was to transiently silence PR-A expression in dopaminergic PC12 cells by specific siRNAs and examine the neurite outgrowth in response to puerarin. Indeed, puerarin failed to induce neurite outgrowth in PR siRNA-transfected cells (Figures 6(a)–6(c)). These results suggest that PR is essential for puerarin to exhibit neurotrophic activity, suggesting that puerarin could potentiate neurite outgrowth via PR signaling. To clarify the dependence of PR, we employed the promoter-reporter system to assay the effects of puerarin on the transcriptional activities of PR. As a result, puerarin markedly induced the transcriptional activity of PR, whereas progesterone specifically induced the transcriptional activity of PR (Figure 6(d)). Moreover, the molecular docking software AutoDock Vina was employed to simulate the binding of the puerarin and progesterone to human PR structure (PDB: 3D90). As shown in Figure 6(e), puerarin and progesterone appeared to bind to the different sites of PR while the residues such as Gln725, Met759, and Arg766 might contribute to the binding. Puerarin and progesterone bind to PR at the binding energy of -8.2 kcal/mol and -10.1 kcal/mol, respectively. Collectively, these results suggested that puerarin attenuated MPTP- and MPP^+^-induced neurotoxicity and potentiated neurite outgrowth via PR signaling.

## 5. Conclusion

The present study demonstrated that puerarin ameliorated MPTP- or MPP^+^-induced neurotoxicity and promoted neurite outgrowth in mouse PD model and cultured primary rat midbrain neurons via PR-dependent mechanism. Our results for the first time suggest that puerarin may mimic progesterone to interact with PR at the cell surface, cytosol, or cell nucleus although the involvement of PGRMC1 is not excluded. Ultimately, puerarin could exhibit neuroprotective and neurotrophic activities via PR signaling (Figure 6(f)). We believe that puerarin may serve as lead compounds for the development of novel PR agonists to treat PD and other neurodegenerative diseases.

## Data Availability

The data were available from the corresponding author upon request.

## Supplementary Materials

Supplementary Materials Figure S1: simplification of Figure 4. Table S1: list of antibodies.
