# Supplementary material for: Botanical Drug Puerarin Promotes Neuronal Survival and Neurite Outgrowth against MPTP/MPP+-Induced Toxicity via Progesterone Receptor Signaling
Source: Oxid Med Cell Longev. 2020 Oct 17;2020:7635291. doi: 10.1155/2020/7635291 (PMC7586160; doi:10.1155/2020/7635291)
Supplement: Supplementary Materials — Figure S1: simplification of Figure 4. Table S1: list of antibodies. [file 7635291.f1.docx]

**Figure S1: Simplification of Fig.4.** Puerarin enhanced the survival of primary midbrain neurons against MPP+-induced neurotoxicity.

**
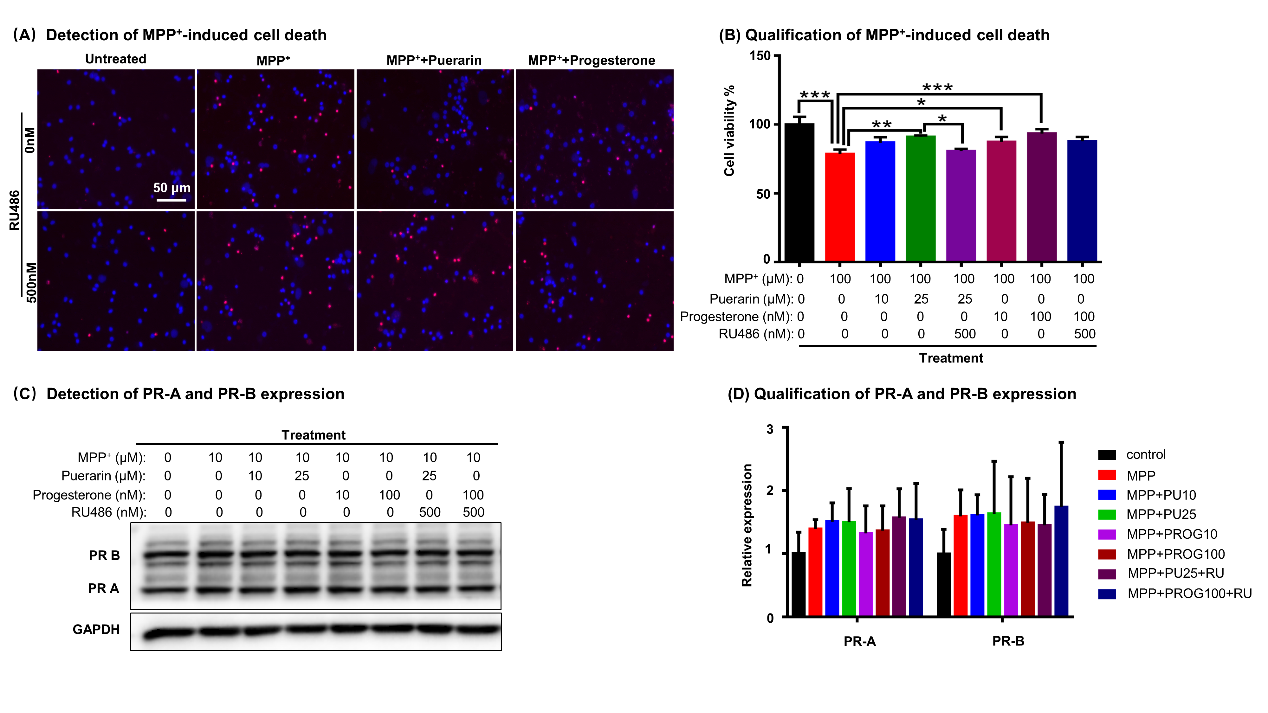
**

**Table S1. List of antibodies**

| **Antibodies** | **supplier** | **species** | **type** | **reference** |
| --- | --- | --- | --- | --- |
| Tyrosine hydroxylase (TH) | Millipore/Biogene | Rabbit | Polyclonal antibody | AB152 |
| PR antibody | Santa cruz | Rabbit | Polyclonal antibody | sc-538 |
| GAPDH (14C10) | Cell signaling | Rabbit | monoclonal antibody | 2118S |
| GAP-43 | Santa cruz | mouse | Monoclonal IgG1 | Sc-17790 |
| Anti-rabbit IgG (whole molecule) | Sigma | Goat | Polyclonal antibody | A0545-1mL |
| Anti-mouse IgG Secondary Antibody, Alexa Fluor 568 | ThermoFisher | Goat | Polyclonal antibody | A-11031 |
| Anti-rabbit IgG Secondary Antibody, Alexa Fluor 594 | ThermoFisher | Goat | Polyclonal antibody | A-11037 |
